# The complicated art of writing the perfect CV

**DOI:** 10.1038/s44319-025-00586-3

**Published:** 2025-09-26

**Authors:** David R Smith

**Affiliations:** University of Western Ontario, London, N6A 5B7 ON Canada

**Keywords:** Careers

## Abstract

Like this author, many scientists obsess about their CVs, constantly upgrading and improving them. But at the end of the day, it is just one element of a convincing application.

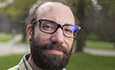

*“You wouldn’t worry so much about what others think of you if you realized how seldom they do.” — Eleanor Roosevelt*

I am embarrassed to admit this, but I used to collect, save, and study other people’s CVs. Indeed, I was a curriculum vitae connoisseur. I have tasted them all: the short and sweet, the overconfident, the muddled mess, and the depressingly impressive. It all started in graduate school, when I was first putting together my own academic CV. What initially seemed like a simple task quickly turned into a daunting endeavour. I kept rearranging everything, trying my best to highlight what little achievements I had. Do I put conference talks before publications? Should I include my undergraduate TAing experience? How do I organize my poster presentations? Is it necessary to put my date of birth? I decided to see how other people were dealing with these questions.

My PhD supervisor, Bob, kindly shared his CV with me and recommended that I peruse other scientists’ research webpages for theirs. Sure enough, many researchers in my field had a link next to their online bios saying: “Download an up-to-date CV.” Within a few hours, I’d compiled a folder with over a dozen CVs of scientists from varying stages of their academic careers. The problem was that those who post their CVs online tend to be high achievers. Before long, I was feeling sorry for myself and my lack of achievements. I walked into Bob’s office with my head hung low and said: “I have this horrible feeling that science is largely a game of my CV is bigger than yours.” He looked at me straight-faced and replied: “You are bang on, Smitty, so you'd better get working on yours.”

Despite my feelings of inadequacy, I dissected each of the CVs I'd downloaded. You can learn a lot about the pathways to success by examining successful people. I started to see trends among the documents, and it was not just *Nature* papers. It was more nuanced, and included things like invited contributions to journals, societal fellowships and awards, international collaborative experiences, as well as specific volunteer and service work at conferences. I was also in awe of the imaginative ways that some had structured their CVs. (No one told me you were allowed to use tables or figures!) Being a first-year PhD student at a little-known university in Nova Scotia, I could not immediately act upon these newfound recipes for success. But at least I now had a template for climbing to the upper echelons of my chosen field. I also had a better understanding of the hard work needed to become a prosperous scientist.

From then on, I became obsessed with my CV. Anything of academic note that I did or accomplished, got added, sometimes even before I did it. It got so bad that when colleagues or peers asked me for assistance, I’d first ask myself: “Is this something I can put on my CV?” If it was not, I would often reply in the negative. By the time I was applying for faculty positions, my CV had taken on a life of its own. Margins, fonts, the use of bold or italics, the spacing between headings and subheadings, the pros and cons of bullet points: nothing was too minute for me to ignore. Things got serious when I started incorporating superscripts and footnotes. It became like a choose your own adventure CV: If you want to see how great Dr. Smith is at publishing papers, please turn to page 5 and the accompanying appendices. For news coverage and media interviews, see page 8. And all for what? To make the world see how productive, brilliant and important I was? If anything, there should have been a superscript on the front page beside my name^(his ambitions exceed his talents)^.

Why are so many academics engrossed with cultivating the perfect CV? It is the standard currency when applying for jobs or grants, so it is understandable why researchers would want to make their CVs the best they can be. But I believe this obsession reflects a deeper underlying preoccupation with hyper productivity, a fixation on more papers, funding, awards, promotions and so on. By constantly updating their CVs, scientists can keep track of all that productivity, like Cub Scouts adding badges to their vests or a Pokémon collector putting another card in the binder. There is also the practical aspect: it is easier to manage and record research and service outputs on the go rather than waiting 6 months and then having to spend hours recalling these past accomplishments, big or small.

My awakening came a few years ago when I was battling colon cancer. Even on sick leave, I was still updating my CV with the outcome of various manuscripts, essays and grants that were in motion. One day I started adding a footnote to my publication entries for 2023: “^*†*^*Note, my productivity for this year was impeded by surgeries and chemotherapy treatments…”* At that moment, something made me pause. This is ridiculous, I thought to myself, I might not have much time to live and here I am using footnotes to account for a small drop in my publication output, as if anyone really gives two hoots about this.

If I think about all the people who truly matter in my life, most if not all have no idea about what is or is not on my CV. My mother, wife and son have never seen my CV and probably never will. Neither have my closest friends or in-laws. In fact, if I tried to show it to any of them, they’d likely roll their eyes and walk away. They do not care that I had thirteen publications in 2022 but only six in 2023 or that my standard operating grant was renewed in 2024. Even my closest colleagues have probably only glanced at my CV in passing, for some mundane annual performance review or to recommend me to a service committee. I still regularly talk with and visit my old PhD supervisor. Bob has never once said: “So, Smitty, how’s that old CV coming a long? I would love to give it a quick once-over.” He would much rather talk about the meaningful memories we shared together in the lab.

Of course, science is still an academic arms race where the CV is the weapon of choice. And mine certainly helped me secure a tenure-track position, promotions, and a handful of awards. But to achieve those milestones, I probably did not need to write the *War and Peace* of resumés, and I certainly could have been a little more generous with my time. To be honest, now when I open the word document Smith_CV_version_107 and add a tenth row to the table titled “Notable Departmental Engagement Activities,” my ego just is not in it anymore. If I could go back to my grad school cubicle when I was downloading that first batch of CVs, I would add a footnote to my thoughts that day: try not to be so self-centered. When mentoring young scientists today, I encourage them to update their CVs but not to spend too much time on them. For example, a CV is an important part of a job application, but so is the cover letter, and, in the final analysis, it is your personality, your knowledge and experiences that count the most, which are not necessarily reflected in the CV.

But old habits die hard, and here I am, writing the final few words of an opinion piece for *EMBO reports*. But I can not think for you, you’ll have to decide whether I’ve already added this latest achievement to page number five.

## Supplementary information

Peer Review File

