# Supplementary material for: The complicated art of writing the perfect CV
Source: EMBO Rep. 2025 Sep 26;26(20):4893–4. doi: 10.1038/s44319-025-00586-3 (PMC12549847; doi:10.1038/s44319-025-00586-3)
Supplement: Supplementary file 1 — Peer Review File [file 44319_2025_586_MOESM1_ESM.pdf]

EMBO reports

The Complicated Art of Writing the Perfect CV

David Smith

Corresponding author(s): David Smith (dsmit242@uwo.ca)

**Review Timeline:**	Submission Date:	16th Sep 25
	Accepted:	16th Sep 25

Editor: Holger Breithaupt

Transaction Report: This article type is not peer reviewed.
